# The explorations of the awareness, contemplation, self-Efficacy, and readiness of advance care planning, and its predictors in Taiwanese patients while receiving hemodialysis treatment

**DOI:** 10.1186/s12904-022-01063-7

**Published:** 2022-10-14

**Authors:** Li-Chen Chen, I-Te Tu, I-Chen Yu, Tao-Hsin Tung, Hsiang-Ping Huang, Yung-Chang Lin, Randal D. Beaton, Sui-Whi Jane

**Affiliations:** 1 Department of Nursing, Chi-Mei Medical Center, Liouying, Tainan, Taiwan; 2 Department of Biomedical Engineering, National Cheng Kung University, Tainan, Taiwan; 3 Division of Nephrology, Department of Internal Medicine, Chi-Mei Medical Center, Liouying, Tainan, Taiwan; 4 Department of Nursing, Chang Gung University of Science and Technology, Tao-Yuan City, Taiwan; 5 Division of Nephrology, Department of Internal Medicine, Chang Gung Memorial Hospital, Lin-Ko, Taiwan; 6 Evidence-based Medicine Center, Taizhou Hospital of Zhejiang Province affiliated to Wenzhou Medical University, Linhai, Zhejiang China; 7 Division of Hematology/Oncology, Department of Internal Medicine, Chang Gung Memorial Hospital, Lin-Ko, Taiwan; 8 Psychosocial & Community Health and Health Services, Schools of Nursing and Public Health, University of Washington, Seattle, USA

**Keywords:** Taiwanese hemodialysis patients, Advance care planning (ACP), Awareness, Contemplation, Self-efficacy, Readiness

## Abstract

**Background:**

End-stage renal disease (ESRD) is a major chronic illness worldwide, and Taiwan reports one of the highest incidence rates of ESRD with 529 cases per million population (pmp). A number of patients with ESRD patients might require lifelong hemodialysis (HD) or peritoneal dialyses (PD). Due to the progression of dialysis, patients are likely to experience other chronic comorbidities, anxiety and depression, frequent hospitalizations, and higher rates of mortality compared to patients with other types of chronic illnesses. As a result, dialysis patients are prone to experience advance care planning (ACP) needs, such as whether they withdraw from receiving dialysis while approaching their end-of-life (EOL). Yet, existing studies have shown that dialysis patients seldom receive timely consultation regarding ACP and there are limited studies examining ACP amongst Taiwan HD patients.

**Purpose:**

The purpose of this study was to examine ACP awareness, contemplation, self-efficacy and readiness; and factors influencing ACP readiness.

**Design:**

This cross-sectional descriptive study with convenience sampling was conducted in the out-patient HD unit at a regional teaching hospital in southern Taiwan. A total of 143 ESRD patients undergoing HD treatments were recruited. A 55-item ACP engagement survey containing the subscales of awareness, contemplation, self-efficacy, and readiness was employed. The data were analyzed with *t*-tests, one-way ANOVAs, Pearson’s correlations and multiple regressions.

**Results:**

The results of our investigation revealed that approximately half of the participants (*n* = 67, 46.9%) were not informed of ACP. Although they reported considering their EOL, medical decisions and desired care, they demonstrated significantly low self-efficacy in discussing ACP (*t*= -5.272, *p* < 0.001). HD duration influenced all four ACP subscales; religious beliefs significantly influenced ACP-self-efficacy and readiness; and marital status, education, and primary decision-maker status significantly influenced ACP-readiness. The predictors of ACP-readiness were high self-efficacy and being the primary decision-maker (Adjusted R^2^ 61%).

**Conclusion:**

Most of the HD patients in this study had low ACP-awareness, contemplation, self-efficacy, and readiness, and most had not completed any ACP-related advance directives (AD). Healthcare professionals should proactively provide HD patients with ACP-related information and answer patients’ and medical decision-makers’ questions in a timely manner, thereby improving the quality of EOL care.

## Background

End-stage renal disease (ESRD) is a major chronic illness, with an estimated incidence rate of 132 cases per million population (pmp) in European countries [1] and 373 cases pmp in the United States [2]. Compared to Western countries, Taiwan reports one of the highest incidence rate of ESRD, with 529 cases pmp [3]. According to Taiwan Annual Report, in 2018 there were 12,346 new dialysis cases and a total of 84,615 dialysis cases, with the prevalence rate of 3,587 pmp [4]. In total, dialysis related healthcare comprises 8.7–9.3% of Taiwan annual national healthcare budget ($1.78 billion) [5]. Due to the characteristics and progression of dialysis, patients undergoing treatment are likely to experience other chronic comorbidities, including hypertension, diabetes, and cardiovascular disease [6], anxiety and depression [7], frequent hospitalization, and higher mortality rates compared to patients with prostate, colorectal, or breast cancer and heart failure [8–10].

Nowadays, medical advancements and improved care for patients undergoing haemodialysis (HD) has led to the adjusted mortality rate decreasing from 192.9/1000 patient-years to 164.6/1000 patient-years; however, the overall mortality rate of patients undergoing HD was 2-2.5 times more than patients with myocardial infarction and cancer [11]. Additionally, up to 30% of dialysis patients in high income countries across North America, Western Europe, and Oceania die from dialysis withdrawal [12]. Thus, patients undergoing dialysis are more likely to encounter ethical and legal concerns related to palliative care and advance care planning (ACP), which involves advance directives (AD), health care agents, and medical decisions for end-of-life (EOL) (i.e., timing of dialysis withdraw, do not resuscitate (DNR) orders and physician orders for life-sustaining treatment). From the perspective of medical care, the assessment of patients’ attitude, preparation and self-efficacy regarding ACP might enhance the quality of EOL care [13–15]. Hence, it is crucial for healthcare teams to provide appropriate EOL care by respecting patients’ autonomy regarding medical decisions in light of their impending incompetency.

Existing research investigations have documented that the fulfilment of patients’ EOL wishes resulted in the amelioration of patients’ anxiety/depression [16, 17] as well as the degree of emotional burden on family decision-makers [18, 19]. Moreover, it can deter conflicts amongst patients and their family members and healthcare providers [20, 21] and reduce unnecessary hospitalization or transferring to intensive care units [22]. Furthermore, healthcare provider-initiated ACP conversations with patients and their surrogates significantly improved patients’ preparation for EOL decision-making [23] and completion of AD [24].

Despite the reported importance of respecting patients’ EOL care, ESRD and dialysis patients still receive limited information regarding ACP [25, 26]. A study in Canada revealed that 90% of patients with chronic renal failure had never discussed their prognoses with their physicians, and 65% of patients were unprepared to make medical decisions while approaching the advanced stages of disease [27]. Similarly, 96% of Australian chronic kidney disease (CKD) patients claimed that they needed more discussion or education on ACP; participants identified that they experienced patient/family discomfort (84%), difficulty engaging families (83%), lack of clinician expertise (83%), health professional discomfort (72%) and language or cultural barriers (65%) [21]. In Spain, although patients undergoing HD preferred to refrain from aggressive treatment during their final stage of illness, only 7.9% of patients completed ADs [28]; furthermore, 95% had little or no knowledge about cardiac resuscitation or mechanical ventilation and 22% believed that this decision should be made by their family members [29]. In addition, it has been found that HD patients’ ACP decision-making is associated with factors such as age [30–32], education level [30, 33], marital status [30, 34], religious beliefs [35], health status or comorbidities [33], duration of HD [13], and frequency of hospitalisation [36].

To ensure patients’ right to make medical decisions and maximise quality of life (QoL) at the EOL, Taiwan established the Hospice Palliative Care Act (HPCA ) in 2000 that EOL patients have rights to choose hospice care rather than aggressive treatment to relieve their physical, psychological and spiritual distress [37]. Considering the limitation of HPCA that was primarily for patients approaching EOL, the Patient Right to Autonomy Act (PRAA) was endorsed in 2020 [38]. The philosophy of PRAA is to encourage competent patients to complete related-AD documents as well as other official written documentation that expresses the patient’s medical preferences [39]. PRAA highlights that patients should receive consultation with a team and that this must take place before the patient encounters the EOL stage, irreversible coma, permeant vegetative status, severe dementia and an unbearable or incurable condition [40]. Presently, only 0.06% of the Taiwanese population has completed ADs [41] whereas 38.2%, 37%, 10%, and 8% of the populations in Canada, United States [42], Netherlands [43], and Germany, respectively [44], have completed ADs. A study conducted by Ma [45] reported that 35.4% of Taiwanese patients were still receiving dialysis prior to death, 12.2% received cardiopulmonary resuscitation (CPR), and 58.9% signed the DNR orders by family members 24h prior to their death. Similarly, a systematic review of ACP in Asia (*n* = 36) found that 32-88% of Asian (Hong Kong, 34–88%; China 32–80%; South Korea 59%) patients perceived the benefits of ACP [46, 47], and yet, the AD completion rate among the general population is merely 0.5% in Hong Kong [47], 10% among Chinese Americans [48]. The low initiation of ACP might be due to the role of Confucianism in Chinese culture, which prioritizes family-centred decision making [49].

To date, ACP-related studies in Western countries primarily focused on chronic pulmonary disease [50, 51], liver disease [52, 53], dementia [54], incurable cancer [55], and CKD [16, 56] or HD/dialysis  [57, 58]. While these studies provide critical insight, these studies do not reflect how ACP is dealt with in Chinese culture, such as in Taiwan, which underscores the need for culturally specific examinations of ACP. Studies based in Taiwan and Singapore [31] have mostly focused on nurse attitudes [59], patients with cancer [60], nursing home residents [61], and COVID-19 patients [62]. Considering the new legislation of the Patient Right to Autonomy Act and infant stage of ACP is still under practiced in Taiwan, it is evident that there remains relatively low awareness of ACP; this is particularly true when comparing ACP in Taiwan to Western countries that have adopted ACP for several years. The limited studies indicate a lack of evidence on the ACP process with other vulnerable patient populations, such as ESRD patients undergoing HD in Taiwan. Thus, the purpose of this descriptive study was to investigate how HD patients perceive ACP and what degrees of their self-efficacy and readiness responding to ACP and what factors influence their ACP decision-making.

## Materials and methods

### Study design and setting

This cross-sectional descriptive study explored awareness, contemplation, self-efficacy and readiness with respect to ACP among patients undergoing HD. Potential participants were purposely sampled from an outpatient HD unit at a regional teaching hospital in southern Taiwan between April and June 2021. Participants were included if they met the following criteria: age ≥ 20 years; ability to understand, speak, and read Chinese; diagnosed with ESRD; and have been received HD for at least 6 months. Patients were excluded from this study if they were experiencing severe fatigue with an intensity ≧ 7 on a 0–10 scale, had deteriorating cognition (disoriented to person, place, or time), clinically diagnosed mental health disorder (e.g., major depression or anxiety) or other physical conditions that prevented them from completing study procedure (e.g., vision or hearing impairment).

This study was reviewed and approved by the Institutional Review Board of Chi Mei Medical Center of Liouying Hospital in Taiwan (IRB # 11,001-L01). The licensed HD nurses would undertake history taking, physical examinations, vital sign checks, and body weight measurements of patients admitted for their regular outpatient HD (three times per week). When the participants sustained stable HD, which can take between 3 and 3.5 h, the first author would approach prospective participants who met the inclusion criteria. Participants were provided with an explanation of the research purpose and procedures. They were given an option to participate and they were informed of their rights with respect to the study. After obtaining written consent, each interview was completed within approximately 25 to 40 min and conducted at the participant’s bedside with the curtain around the participant’s bed and posted a sign “In Session” to minimize distractions and ensure privacy. During the entire interview, we would monitor the patient’s blood pressure and orientation status; and stop interview if the patient reported with any discomfort, to enhance the quality of interview.

## Instruments

### Demographic information

Participants’ demographic information included age, gender, religion, marital status, education level, and medical information including details of previous ESRD treatment, duration of HD and comorbidities.

### Awareness, contemplation, self-efficacy, and readiness of ACP (ACSR-ACP)

The ACSR-ACP is a 55-item measure originally developed from Sudore et al. [63] Advance Care Planning Engagement Survey. The measure comprises four subscales: knowledge (6 items), contemplation (17 items), self-efficacy (13 items), and readiness (19 items) [64]. Sudore et al. [63] used social cognitive and behaviour change theories to develop the original 82-item ACP-ES with 4 ACP domains (surrogate decision-makers, quality of life, flexibility in surrogate decision-making, and asking doctors questions) with a focus on four behavioural change constructs (awareness, contemplation, self-efficacy, and readiness). After factor analysis, the original ACP-ES was revised to a 55-item version, with a Cronbach’s alpha of 0.97 and 58.5–68.8% of the variance explained [64]. It has been used in several interventional studies of chronic illness [65] and dialysis populations [63, 65]. These subscales are scored on a 5-point Likert scale, with answers ranging from 5 (extremely, a lot, or I have already done it) to 1 (not at all, never, or I have never thought about it). Adapted cut-off points for knowledge, attitudes and practice behaviors of ACP for nurses were made in our previous ACP study [59], a score of 3 (neutral) was used as a cut-off point for differentiating positive (more than 3) or negative (below 3) tendency. A higher score indicated more frequent ACP or more confidence or readiness with respect to ACP.

In addition to obtaining permission to use the ACSR-ACP tool from Dr. Sudore, we also obtained the established reliable and valid Chinese version of the self-efficacy and readiness subscores of ACSR-ACP tool conducted in Hong Kong and approved by Sudores team [66], with Cronbach’s alphas of 0.97 and 0.94, respectively. As this study was cross-cultural in nature, the technique of translation and back-translation was employed to ensure the cultural coherence between source (original), target (Chinese), and back-translated versions of the subscore of awareness and contemplation of ACSR-ACP. The instrument was translated into Chinese by the first author, afterwards another biolinguistics expert independently translated this translated version back into English (back-translation), and a second biolinguistics expert examined the equivalence of this back-translation version. The overall Cronbach’s alpha of the ACSR-ACP in this study was 0.97 (*awareness 0.91, contemplation 0.93, self-efficacy, 0.93, and readiness* 0.90) and the content validity index (CVI) of subscore of ACSR-ACP ranged from 0.86 to 1 by three researcher specializing in nursing, nephrology, and medicine.

## Data analyses

SPSS 20.0 software was employed for descriptive (i.e., percentages, means, and standard deviations [SDs]) and inferential analyses (i.e., independent sample *t* tests, one-way analysis of variance, ANOVA, Scheffe’s comparisons, and Pearson correlation). Multiple regressions were used to investigate predictors of ACP. The sample size was determined based on nine independent variables, a moderate effect size of 0.13 proposed by Cohen and Kotrl for multiple regressions [67, 68], an alpha of 0.05, a power of 80%, and an estimated 10% refusal rate, accounting for a total of 143 participants.

## Results

### Sample characteristics

Of the 177 patients screened, 143 (80.7%) met the eligibility criteria. The other 34 patients were deemed ineligible to participate and excluded from the study for a variety of reasons including the following: (a) receiving HD less than 6 months (54%); (b) the presence of physical distress (e.g., severe fatigue or confusion) (44%); and (c) having other exclusion criteria (e.g., loss of hearing or communication difficulties 2%). Of those eligible to enroll in the study, none declined to participate. In total, 143 patients participated, with a mean age of 63.1 (SD = 9.98, 30–90 years). The majority were men (*n* = 89, 62.2%), married (*n* = 105, 73.4%), had Taoist or Buddhist religious beliefs (*n *= 86, 60.1%), and had junior or high school education levels (*n* = 67, 46.9%). The average duration of HD was 73.1 months (SD = 51.25, 6-230 months). The primary medical decision-makers were mostly the patients themselves (n = 103, 72.0%), followed by spouses (*n* = 21, 14.7%) and parents, brothers, sisters, and children (*n* = 19, 13.3%) (Table 1).

**Table 1:** Demographic characteristics of the sample (*N* = 143)

Variable	*N* (%)	Mean ± SD
Age		63.1 ± 9.98
≦ 60 years	53(37.1)	
≧ 61 years	90(62.9)	
Gender		
Male	89(62.2)	
Female	54(37.8)	
Religion		
Catholic or Christian	12(8.4)	
Folk beliefs or Other	45(31.5)	
Buddhism or Taoism	86(60.1)	
Marital status		
Single	17(11.9)	
Married	105(73.4)	
Divorced or Widowed	21(14.7)	
Education		
Uneducated or Primary school	52(36.4)	
Junior high school	34(23.7)	
High school	33(23.1)	
College/University or above	24(16.8)	
Duration of hemodialysis (month)		73.1 ± 51.25
6–12(1 year)	8(5.6)	
13–60(5years)	60(42.0)	
61–120(10years)	44(30.8)	
121–230(19.2years)	31(21.7)	
Past dialysis history		
Hemodialysis	134(93.7)	
Peritoneal dialysis	8(5.6)	
Kidney transplant	1(0.7)	
Primary medical decision makers		
Self	103(72.0)	
Spouse	21(14.7)	
Parent or Sibling/Child	19(13.3)	
Disease comorbidity score^a^		3.92 ± 1.73
≦ 2	39(27.3)	
2–5	80(55.9)	
≧ 6	24(16.8)	

Note: ^a^Charlson cormorbidity index was measured with 17 items, scoring with 0 (No) and 1 (Yes)

### ACSR-ACP

Scores for the items on the ACP-awareness subscale (6 items) ranged from 2.42 to 2.66 (2.52 ± 1.66), with an overall mean of 15 (SD = 8.19) out of a total score of 30 (*t*=-4.282, p < 0.001). The item with the highest score was “How well informed are you about who can be a medical decision maker?” (2.66 ± 1.72), followed by “How well informed are you about the types of decisions that a medical decision maker may have to make for you in the future?” (2.50 ± 1.64). The item with the lowest score was “How well informed are you about the types of questions you can ask your doctor that will help you make a good medical decision?” (2.42 ± 1.54) (Table 2). Amongst the respondents, 44.8-47.6% reported “not at all” informed (1 score) on the subscale of ACP-awareness.

**Table 2:** Mean and Standard Deviation of Awareness, Contemplation, Self-Efficacy and Readiness of Advance Care Planning (*N* = 143)

Items		Mean ± SD	*t*	*p*
***Advance Care Planning – Awareness (6 items)***				
1	How well informed are you about who can be a medical decision maker?	2.66 ± 1.72	-2.323	0.022
2	How well informed are you about what makes someone a good medical decision maker?	2.45 ± 1.61	-4.096	< 0.001
3	How well informed are you about the types of decisions that a medical decision maker may have to make for you in the future?	2.50 ± 1.64	-3.663	< 0.001
4	How well informed are you about what it means to give a medical decision maker flexibility to make future decisions?	2.48 ± 1.66	-3.716	< 0.001
5	How well informed are you about the different amounts of flexibility a person can give their medical decision maker?	2.49 ± 1.66	-3.657	< 0.001
6	How well informed are you about the types of questions you can ask your doctor that will help you make a good medical decision?	2.42 ± 1.54	-4.493	< 0.001
	Subtotal score	15 ± 8.19	-4.382	< 0.001
***Advance Care Planning – Contemplation (17 items)***			*t*	*p*
7	How much have you thought about who your medical decision maker should be?	3.57 ± 1.73	3.954	< 0.001
8	How much have you thought about asking someone to be your medical decision maker?	3.27 ± 1.81	1.758	0.081
9	How much have you thought about talking with your doctors about who you want your medical decision maker to be?	1.38 ± 1.05	-18.439	< 0.001
10	How much have you thought about talking with your other family and friends about who you want your medical decision maker to be?	2.98 ± 1.85	− 0.136	0.892
11	How much have you thought about whether or not certain health situations would make your life not worth living?	3.92 ± 1.44	7.626	< 0.001
12	How much have you thought about talking with your decision maker about whether or not certain health situations would make your life not worth living?	3.06 ± 1.82	0.413	0.680
13	How much have you thought about talking with your doctors about whether or not certain health situations would make your life not worth living?	1.32 ± 1.01	-19.847	< 0.001
14	How much have you thought about talking with your other family and friends about whether or not certain health situations would make your life not worth living?	2.98 ± 1.80	− 0.140	0.889
15	How much have you thought about the care you would want if you were very sick or near the end of life?	3.87 ± 1.47	7.101	< 0.001
16	How much have you thought about talking with your decision maker about the care you would want if you were very sick or near the end of life?	2.92 ± 1.84	− 0.500	0.618
17	How much have you thought about talking with your doctors about the care you would want if you were very sick or near the end of life?	1.21 ± 0.79	-26.934	< 0.001
18	How much have you thought about talking with your other family and friends about the care you would want if you were very sick or near the end of life?	2.87 ± 1.82	− 0.871	0.385
19	How much have you thought about the amount of flexibility you would want to give your medical decision maker?	2.68 ± 1.75	-2.197	0.030
20	How much have you thought about talking with your medical decision maker about how much flexibility you want to give them?	2.64 ± 1.75	-2.484	0.014
21	How much have you thought about talking with your doctor about how much flexibility you want to give your decision maker?	1.31 ± 0.98	-20.530	< 0.001
22	How much have you thought about talking with other family and friends about how much flexibility you want to give your decision maker?	2.50 ± 1.67	-3.548	0.001
23	How much have you thought about questions you will ask your doctor to help make good medical decisions?	2.36 ± 1.54	-4.995	< 0.001
	Subtotal score	44.8 ± 18.5	-3.980	< 0.001
***Advance Care Planning - Self-Efficacy(13items)***			*t*	*p*
24	How confident are you that today you could ask someone to be your medical decision maker?	3.13 ± 1.80	0.838	0.403
25	How confident are you that today you could talk with your doctors about who you want your medical decision maker to be?	1.70 ± 1.37	-11.320	< 0.001
26	How confident are you that today you could talk with your other family and friends about who you want your medical decision maker to be?	2.90 ± 1.82	− 0.688	0.492
27	How confident are you that today you could talk with your decision maker about whether or not certain health situations would make your life not worth living?	2.99 ± 1.82	− 0.092	0.927
28	How confident are you that today you could talk with your doctors about whether or not certain health situations would make your life not worth living?	1.45 ± 1.15	-16.085	< 0.001
29	How confident are you that today you could talk with your other family and friends about whether or not certain health situations would make your life notworth living?	2.97 ± 1.80	− 0.186	0.853
30	How confident are you that today you could talk with your decision maker about the care you would want if you were very sick or near the end of life?	2.99 ± 1.82	− 0.092	0.927
31	How confident are you that today you could talk with your doctors about the care you would want if you were very sick or near the end of life?	1.48 ± 1.08	-16.798	< 0.001
32	How confident are you that today you could talk with your other family and friends about the care you would want if you were very sick or near the end of life?	3.04 ± 1.76	0.285	0.776
33	How confident are you that today you could talk with your decision maker about how much flexibility you want to give them?	2.85 ± 1.76	-1.000	0.319
34	How confident are you that today you could talk with your doctors about how much flexibility you want to give your medical decision maker?	1.57 ± 1.27	-13.496	< 0.001
35	How confident are you that today you could talk with your other family and friends about how much flexibility you want to give your medical decision maker?	2.70 ± 1.76	-2.043	0.043
36	How confident are you that today you could ask the right questions of your doctor to help make good medical decisions?	2.35 ± 1.56	-4.993	< 0.001
	Subtotal score	32.1 ± 15.6	-5.272	< 0.001
***Advance Care Planning - Readiness (19 items)***			*t*	*p*
37	How ready are you to formally ask someone to be your medical decision maker?	2.27 ± 1.73	-5.070	< 0.001
38	How ready are you to talk with your doctors about who you want your medical decision maker to be?	1.32 ± 1.03	-19.449	< 0.001
39	How ready are you to talk with your other family and friends about who you want your medical decision maker to be?	2.85 ± 1.29	-1.431	0.155
40	How ready are you to sign official papers naming a person or group of people to make medical decisions for you?	1.36 ± 0.96	-20.475	< 0.001
41	How ready are you to decide whether or not certain health situations would make your life not worth living?	2.62 ± 1.61	-2.807	0.006
42	How ready are you to talk to your decision maker about whether or not certain health situations would make your life not worth living?	2.23 ± 1.65	-5.584	< 0.001
43	How ready are you to talk to your doctors about whether or not certain health situations would make your life not worth living?	1.16 ± 0.69	-31.946	< 0.001
44	How ready are you to talk to your other family and friends about whether or not certain health situations would make your life not worth living?	2.27 ± 1.58	-5.521	< 0.001
45	How ready are you to sign official papers putting your wishes in writing about whether or not certain health situations would make your life not worth living?	1.45 ± 1.08	-17.204	< 0.001
46	How ready are you to decide on the medical care you would want if you were very sick or near the end of life?	2.77 ± 1.62	-1.706	0.090
47	How ready are you to talk to your decision maker about the kind of medical care you would want if you were very sick or near the end of life?	2.36 ± 1.68	-4.579	< 0.001
48	How ready are you to talk to your doctors about the kind of medical care you would want if you were very sick or near the end of life?	1.17 ± 0.63	-34.897	< 0.001
49	How ready are you to talk to your other family and friends about the kind of medical care you would want if you were very sick or near the end of life?	2.40 ± 1.59	-4.515	< 0.001
50	How ready are you to sign official papers putting your wishes about the kind of medical care you would want if you were very sick or near the end of life?	1.48 ± 1.09	-16.698	< 0.001
51	How ready are you to talk to your decision maker about how much flexibility you want to give them?	2.07 ± 1.55	-7.174	< 0.001
52	How ready are you to talk to your doctors about how much flexibility you want to give your decision maker?	1.20 ± 0.70	-30.616	< 0.001
53	How ready are you to talk to your other family and friends about how much flexibility you want to give your medical decision maker?	2.00 ± 1.48	-8.093	< 0.001
54	How ready are you to sign official papers to put your wishes in writing about how much flexibility to give your decision maker?	1.31 ± 0.92	-21.847	< 0.001
55	How ready are you to ask your doctor questions to help you make a good medical decision?	1.35 ± 0.82	-24.190	< 0.001
	Subtotal score	35.6 ± 14.6	-17.515	< 0.001

The scores for the items on the ACP-contemplation subscale (17 items) ranged from 1.21 to 3.92 (2.64 ± 1.54), with an overall mean of 44.8 (SD = 18.5) out of a total score of 85 (*t*=-3.980, p < 0.001). The item with the highest score was “How much have you thought about whether or not certain health situations would make your life not worth living?” (3.92 ± 1.44), followed by “How much have you thought about the care you would want if you were very sick or near the end of life?” (3.87 ± 1.47). The item with the lowest score was “How much have you thought about talking with your doctors about the care you would want if you were very sick or near the end of life?” (1.21 ± 0.79).

The scores for the items on the ACP-self-efficacy subscale (13 items) ranged from 1.45 to 3.13 (2.47 ± 1.6), with an overall mean of 32.1 (SD = 15.6) out of a total score of 65 (*t*=-5.272, p < 0.001). The item with the highest score was “How confident are you that today you could ask someone to be your medical decision maker?” (3.13 ± 1.80), and the item with the lowest score was “How confident are you that today you could talk with your doctors about whether or not certain health situations would make your life not worth living?” (1.45 ± 1.15).

The scores for the items on the ACP readiness subscale (19 items) ranged from 1.16 to 2.85 (1.88 ± 1.25), with an overall mean of 35.6 (SD = 14.6) out of a total score of 95 (*t*=-17.515, p < 0.001). The item with the highest score was “How ready are you to talk with your other family and friends about who you want your medical decision maker to be?” (2.85 ± 1.29), and the item with the lowest score was “How ready are you to talk to your doctors about whether or not certain health situations would make your life not worth living?” (1.16 ± 0.69).

### Differences in ACP responses with respect to patient characteristics and predictive factors

Five factors were identified as affecting ACP, including HD duration, religion, marital status, educational level, and being the main decision-maker. Results indicated that patients who had received HD for over 61 months, compared to patients with an HD duration of 60 months or less, showed greater ACP-awareness 16.40 ± 8.20 vs. 13.46 ± 7.59, *p* = 0.031; ACP-contemplation 47.79 ± 19.53 vs. 41.60 ± 16.82, *p* = 0.045; ACP-self-efficacy 35.17 ± 16.71 vs. 28.74 ± 13.67, *p* = 0.013, and ACP-readiness 39.13 ± 16.61 vs. 31.76 ± 10.84, *p* = 0.002 (Table 3).

**Table 3:** The Differences of Characteristics in Awareness, Contemplation, Self-Efficacy and Readiness of Advance Care Planning (*N* = 143)

		Awareness		Contemplation		Self-Efficacy				Readiness	
variable	*n*	M ± SD	*p* value	M ± SD	*p* value	M ± SD			*p* value	M ± SD	*p* value
Age			0.072		0.400				0.544		0.853
≦60 years	53	16.60 ± 8.22		46.55 ± 18.67		33.15	±	15.98		35.92 ± 15.39	
≧61 years	90	14.06 ± 8.07		43.84 ± 18.41		31.50	±	15.47		35.46 ± 14.19	
Gender			0.757		0.791				0.471		0.715
Male	89	15.07 ± 8.07		44.32 ± 18.36		31.11	±	14.83		35.17 ± 13.23	
Female	54	14.89 ± 8.46		45.72 ± 18.84		33.76	±	16.87		36.39 ± 16.70	
Religion			0.108		0.089				**0.006** ^**^		**0.001** ^**^
Buddhism or Taoism	86	15.90 ± 7.69		46.99 ± 17.01		35.01	±	14.55		38.84 ± 15.21	
Other	57	13.65 ± 8.78		41.61 ± 20.24		27.74	±	16.28		30.79 ± 12.22	
Marital status			0.259		0.054				0.070		**0.028** ^*^
Single/Widowed	38	13.71 ± 8.57		39.89 ± 19.31		28.18	±	15.54		31.18 ± 13.19	
Married	105	15.47 ± 8.04		46.64 ± 17.94		33.53	±	15.48		37.24 ± 14.80	
Education			0.183		0.084				0.157		**0.026** ^*^
Below Junior high school	86	14.26 ± 8.14		42.67 ± 17.67		30.60	±	15.07		33.43 ± 12.03	
High school or above	57	16.12 ± 8.20		48.12 ± 19.35		34.39	±	16.30		38.95 ± 17.37	
Time since receiving hemodialysis(month)			**0.031** ^*^		**0.045** ^*^				**0.013** ^*^		**0.002** ^*^
≦60 (5 years)	68	13.46 ± 7.95		41.60 ± 16.82		28.74	±	13.67		31.76 ± 10.84	
≧61(5.1 years)	75	16.40 ± 8.20		47.79 ± 19.53		35.17	±	16.71		39.13 ± 16.61	
Past dialysis history			0.301		0.054				0.21		0.244
Hemodialysis	134	14.81 ± 8.09		44.08 ± 18.11		31.7	±	15.44		35.13 ± 13.93	
Peritoneal dialysis	8	18.00 ± 10.31		58.50 ± 21.73		39.5	±	18.82		44.88 ± 22.84	
Primary medical-decision makers			0.428		0.913				0.242		**0.001** ^**^
Self	103	15.34 ± 7.91		44.95 ± 18.51		33.07	±	16.16		38.17 ± 15.63	
Others	40	14.13 ± 8.92		44.58 ± 18.68		29.65	±	14.03		29.10 ± 8.70	
Disease comorbidity			0.584		0.555				0.088		0.063
≦4	98	14.74 ± 8.43		44.22 ± 19.26		30.60	±	15.20		34.09 ± 14.31	
≧5	45	15.56 ± 7.70		46.20 ± 16.82		35.40	±	16.19		38.98 ± 14.79	

Note.^*^P < 0.05, ^**^P < 0.01

Participants with Buddhist or Taoist beliefs had significantly higher ACP-self-efficacy (35.01 ± 14.55) and ACP-readiness (38.84 ± 15.21) than those with other or no religious beliefs (27.74 ± 16.28, *p* = 0.006 and 30.79 ± 12.22, *p* = 0.001, respectively). In addition, the ACP readiness scores were significantly different based on marriage status, education level, and being the decision-maker. Specifically, married patients (37.24 ± 14.80) showed significantly higher ACP-readiness scores than those of single or widowed patients (31.18 ± 13.19, *p* = 0.028). Patients with more than junior high school level education (38.95 ± 17.37) had significantly higher ACP-readiness scores than patients with less than junior high school level education (33.43 ± 12.03, *p* = 0.026). Patients who were their own primary medical decision-makers (38.17 ± 15.63) scored significantly higher than patients with family members who were the primary decision-makers (29.10 ± 8.70, *p* = 0.001).

All four subscales of ACP (awareness, contemplation, self-efficacy, and readiness) were significantly and positively correlated. For example, when ACP-readiness was higher, ACP-awareness (γ = 0.53, *p* < 0.001), ACP-contemplation (γ = 0.73, *p* < 0.001), and ACP-self-efficacy (γ = 0.78, *p* < 0.001) were higher (Table 4).

**Table 4:** Correlations between Awareness, Contemplation, Self-Efficacy and Readiness of Advance Care Planning (*N* = 143)

Correlation coefficient	Readiness	Awareness	Contemplation	Self-Efficacy
Readiness	-			
Awareness	0.53^***^	-		
Contemplation	0.73^***^	0.77^***^	-	
Self-Efficacy	0.78^***^	0.70^***^	0.86^***^	-

Note.^***^p < 0.001

According to the social cognitive and behaviour change theories used in the original 55-item ACPEs proposed by Sudore et al. [63], behaviour (readiness) was theoretically influenced by cognitive event (awareness), intention (contemplation), and self-efficacy, thus, the authors in this study made readiness a dependent variable. Stepwise multiple regression analyses were employed to determine the predictors of ACP-readiness. The demographic variables that were significantly influential in the univariate analysis (i.e., religious beliefs, marital status, education level, HD duration, and primary medical decision-maker) and ACP-awareness, contemplation and self-efficacy were set as independent variables (ID). The results of regressions revealed an overall explained variation in ACP-readiness of 61.0% (*F* = 111.962, *p* < 0.001). Readiness was higher when self-efficacy (β = 0.736, *p* < 0.001) was higher and when patients were their own primary medical decision-maker (β = −0.207, *p* < 0.001 (Table 5).

**Table 5:** Predictors of the Readiness of Advance Care Planning (*N* = 143)

Model	Standardized Coefficient	*t*	*P*	Adjusted R^2^	*F*
	*Beta*				
Constant	15.423	8.300	< 0.001	0.61	111.962
Primary medical-decision makers	-0.207	-3.935	< 0.001		
Self-efficacy	0.736	13.979	< 0.001		
Religion	− 0.042	-0.744	0.458		
Marital status	0.079	1.489	0.139		
Education	0.074	1.401	0.163		
Time since receiving hemodialysis	0.067	1.236	0.219		
Awearness	− 0.080	-1.081	0.281		
Contemplation	0.162	1.578	0.117		

## Discussion

This is the first descriptive study to evaluate ACP-awareness, contemplation, self-efficacy, and readiness and to identify the predictors of ACP-readiness in 143 Taiwanese patients undergoing HD. This investigation adds to the literature, which has primarily investigated Western populations, by exploring ACP understanding among patients with HD in Taiwan. The results of our investigation revealed that approximately half of the participants were not informed of ACP. Although they reported considering their EOL, medical decisions and desired care, they demonstrated low confidence in discussing ACP. With respect to differences related to personal characteristics, HD duration influenced all four ACP subscales; religious beliefs significantly influenced ACP-self-efficacy and readiness; and marital status, education, and primary decision-maker status significantly influenced ACP-readiness. The predictors of ACP-readiness were high self-efficacy and being the primary decision-maker.

In terms of ACP-awareness, approximately 50% of the participants in this study indicated that they were “not at all,” informed of ACP. The notable number of patients with little understanding of ACP were consistent to related studies [27, 49, 59]. The phenomena of being less informed of ACP amongst Chinese participants might be explained by aspects of Chinese culture. Namely, Chinese culture is heavily dominated by Confucianism, which emphasizes self-discipline and not causing burden to others. As such, it is considered appropriate to refrain from exploring personal issues and to even hide bad news from patients [69]. As a result, Chinese families may be reluctant to initiate ACP related conversations; instead, it is perceived as more appropriate to carry out decision-making for patients in a family discussion. In a previous study, for instance, it was found that Taiwanese nursing personnel rarely proactively discussed ACP with their patients due to not being sure when it would be appropriate to initiate such conversations [59]. The major barriers amongst health professionals promoting ACP are attributed to lack of education and confidence as well as insufficient time to discuss ACP with patients [46, 57, 70]. Other studies also suggest considering patients’ value, culture and preferences [58] and to include family members in the ACP discussion process. Other factors associated with institutional policies, cultural and ethnic influences, and information provided by healthcare professionals could also lead to less awareness of ACP among patients [71]. That said, even patients who were less informed about ACP reported thinking about ACP (ACP-contemplation) “once or twice” or “a few times” and considered their EOL medical decisions more than once. Although many patients had briefly engaged with ACP-contemplation, more than 80% of patients indicated that they had never considered discussing EOL care issues with their physicians. The above results highlight the importance of the role and function of healthcare providers in actively initiating ACP-related conversations with patients.

On the ACP-self-efficacy subscale, most participants in this study indicated that they were “a little” to “somewhat” confident in discussing ACP with medical decision-makers, family members and friends. However, most reported that they were “not at all” or “a little” confident in discussing topics with their doctors, indicating that they did not have the confidence to discuss EOL or desired care with their physicians when their conditions were severe. A study of patients with end-stage respiratory disease revealed that the percentage of patients who discussed ACP with their physicians (30.3%) was significantly lower than that of patients who discussed it with their family members (54.2%) [50]. This phenomenon is also reflected in a systematic review of Asian healthcare professionals, which noted that although healthcare agents considered ACP to be essential, they lacked the knowledge and skills required to complete ACP and were, therefore, uncomfortable doing so [69]. In addition, healthcare agents were worried about potential conflicts with the patient’s family and subsequent legal consequences, which prevented them from initiating ACP. This may be a reason why few patients were willing to discuss ACP with their physicians. As a result, it was not surprising that the mean score of the ACP-readiness subscale in this study was less than 3 points, indicating that most participants were not ready to complete any ACP-related tasks within the following 6 months (“I am thinking about doing it in the next 6 months”). Among the nine items, patients gave lower scores for “How ready are you to talk to your doctor…” and “How ready are you to sign official papers…” (˂1.5 points). Approximately 90% of the patients had never thought about discussing ACP with their physicians and were not ready to sign any ACP-related documents. Another study also demonstrated that patients rarely discussed EOL-related topics with their physicians, with 65% of the included patients reporting that they were not ready to discuss their preferred EOL care [27], indicating low ACP readiness. Interfere.

With respect to the predictors of ACP-readiness, the results of the Pearson’s correlation analyses revealed that awareness, contemplation, self-efficacy, and readiness of ACP were significantly and positively correlated with each other. Moreover, the results of ANOVA revealed that HD duration, marital status, education level, religious beliefs, and primary medical decision-makers significantly affected ACP-readiness. In the multiple regression analyses, however, only the self-efficacy and primary medical decision-makers were significant predictors (explanatory power of 61%), indicating that patients’ ACP-readiness increased the most when their self-efficacy was high and when they had a spouse, parent, or child assisting them in medical decision-making. In other words, patients with high level of self-efficacy demonstrated greater confidence in communicating ACP-related issues with their families or healthcare professionals, thereby improving their ACP-readiness. These results were consistent with Liu et al. [66] findings, which reported that self-efficacy had a significant and strong correlation with readiness (*r* = 0.809; *P* < 0.01). Considering the uniqueness that self-efficacy plays in the readiness of ACP, future investigations might develop interventional programs to empower dialysis patients’ confidence [16].

Finally, in this study it was found that when the patients were their own primary medical decision-makers, they had higher ACP-readiness derived from the original independent sample *t* test results. Yet, family members assisting in decision-making resulted in higher ACP-readiness from the multiple regression results. Our findings might imply that although patients have higher readiness when they are their own primary decision-makers, assistance in medical decision-making from a spouse, parent, or child could enhance the patients’ ACP-readiness. Indeed, most patients in this study experienced ACP-readiness between the precontemplation stage and the contemplation stage of the stages of change theory. Nevertheless, the ACP-readiness scores in our study were relatively low, which indicates the need for additional steps to be taken to prepare HD patients for ACP.

### Conclusion

In conclusion, this descriptive study revealed that approximately half of the HD participants were not informed of ACP and demonstrated low confidence in discussing ACP. The awareness, contemplation, self-efficacy, and readiness of ACP were significantly and positively correlated. In terms of factors affecting ACP, HD duration influenced all four ACP subscales; religious beliefs significantly influenced ACP self-efficacy and readiness; and marital status, education, and primary decision-maker status significantly influenced ACP-readiness. The predictors of ACP-readiness were high self-efficacy and being the primary decision-maker.

### Clinical implications

To improve the EOL stage for HD patients, healthcare professionals should proactively offer ACP-related information and answer questions from patients and their medical decision-makers in a timely manner. The healthcare system might develop a comprehensive ACP-promotion model to enhance healthcare professionals’ confidence in discussing EOL care preferences with patients. For instance, the development of educational programs on communication and cultural sensitivity training relating to ACP may be worthwhile [72]. Namely, efforts to cultivate patients and decision-makers ACP-awareness, contemplation, self-efficacy, and readiness, can improve the quality of EOL care and assist patients in achieving their palliative care goals. In clinical practice, healthcare profession might also consider to offer prognostic information to their patients under an informed consent for dialysis model.

## Limitations and recommendations

Given that study employed a cross-sectional design. As such, it only included patients undergoing HD in a teaching hospital in southern Taiwan. Additionally, our study deployed purposive sampling to recruit a relatively small sample size (*N* = 143). Indeed, a more diverse and wider range of participants might have yielded new insights. It was also noteworthy that our study did not take participants’ previous exposure to ACP practice into account; previous experiences with ACP might have impacted participants’ responses to our survey. In addition, a substantial body of literature demonstrated that hemodialysis induced significant reductions in cerebral blood flow (CBF) in HD patients [73], and the HD-imposed cerebral ischemia was correlated with decreased executive cognition function [74]; thus the potential impact of the change of CBF on cognition during HD session could not fully be ignored. Lastly, the limitation in this study was regarded with the privacy of interview, for all interviews were not conducted at private rooms that might interfere with participant’s willingness to respond to ACP.

The above limitations impact the generalizability of study findings; therefore, the results drawn from this study must be interpreted with caution. Moreover, these results cannot be used to make inferences and predictions regarding long-term causality. To improve the effectiveness of the clinical implementation of ACP, future studies might consider employing in-depth qualitative interviews on ACP-related topics to identify barriers to ACP. In addition, family members (or medical decision-makers) can be included in future studies. In doing so, it may be possible to examine factors associated with ACP-awareness, contemplation, self-efficacy, and readiness, to understand whether the stages of behavioural change are consistent among patients and family members. To improve the privacy of interview and minimize the potential impact of the change of CBF during HD session, future study might consider initiating ACP-related interviews prior to HD at private rooms. Finally, such research may enable healthcare providers to better assist patients and their families in upholding the patient’s autonomy in the ACP process.

## Data Availability

The datasets generated and analysed during the current study are not publicly available due to the confidentiality of participants’ personal information and interview responses but are available from the corresponding author on reasonable request.

## Declarations

**Publisher’s Note**.

Springer Nature remains neutral with regard to jurisdictional claims in published maps and institutional affiliations.
